# Axl‐inhibitor bemcentinib alleviates mitochondrial dysfunction in the unilateral ureter obstruction murine model

**DOI:** 10.1111/jcmm.16769

**Published:** 2021-07-05

**Authors:** August Hoel, Tarig Osman, Fredrik Hoel, Hassan Elsaid, Tony Chen, Lea Landolt, Janka Babickova, Karl Johan Tronstad, James B. Lorens, Gro Gausdal, Hans‐Peter Marti, Jessica Furriol

**Affiliations:** ^1^ Department of Clinical Medicine University of Bergen Bergen Norway; ^2^ Department of Biomedicine University of Bergen Bergen Norway; ^3^ Faculty of Medicine Institute of Molecular Biomedicine Comenius University in Bratislava Bratislava Slovakia; ^4^ BerGenBio ASA Bergen Norway; ^5^ Department of Biomedicine Center for Cancer Biomarkers University of Bergen Bergen Norway; ^6^ Department of Medicine Haukeland University Hospital Bergen Norway

**Keywords:** AXL inhibition, bemcentinib, mitochondria, oxidative stress, renal fibrosis, UUO model

## Abstract

Renal fibrosis is a progressive histological manifestation leading to chronic kidney disease (CKD) and associated with mitochondrial dysfunction. In previous work, we showed that Bemcentinib, an Axl receptor tyrosine kinase inhibitor, reduced fibrosis development. In this study, to investigate its effects on mitochondrial dysfunction in renal fibrosis, we analysed genome‐wide transcriptomics data from a unilateral ureter obstruction (UUO) murine model in the presence or absence of bemcentinib (n = 6 per group) and SHAM‐operated (n = 4) mice. Kidney ligation resulted in dysregulation of mitochondria‐related pathways, with a significant reduction in the expression of oxidative phosphorylation (OXPHOS), fatty acid oxidation (FAO), citric acid cycle (TCA), response to reactive oxygen species and amino acid metabolism‐related genes. Bemcentinib treatment increased the expression of these genes. In contrast, AKT/PI3K signalling pathway genes were up‐regulated upon UUO, but bemcentinib largely inhibited their expression. At the functional level, ligation reduced mitochondrial biomass, which was increased upon bemcentinib treatment. Serum metabolomics analysis also showed a normalizing amino acid profile in UUO, compared with SHAM‐operated mice following bemcentinib treatment. Our data suggest that mitochondria and mitochondria‐related pathways are dramatically affected by UUO surgery and treatment with Axl‐inhibitor bemcentinib partially reverses these effects.

## 1 INTRODUCTION

Chronic kidney disease (CKD) is a major public health problem, with increasing incidence worldwide.^1^ In 2017, the worldwide prevalence of CKD was 9.1% accounting for around 697.5 million cases, and 1.2 million patients died from CKD. Furthermore, between 1990 and 2017, the global all‐age mortality rate from CKD increased by 41.5% and is expected to continue rising as a result of increased prevalence of cardiovascular diseases and diabetes mellitus, and to increased longevity.^1^

Chronic kidney disease includes a group of diseases characterized by progressive loss of renal function accompanied by increased tissue fibrosis. In general, all progressive forms of CKD show similar fibrosis manifestations suggesting a common pathogenic pathway.^2^ However, although innovative potential therapeutic approaches for CKD have been proposed, over the last 20 years, no new drug has been approved to specifically prevent CKD or to improve kidney function.^3^, ^4^ Furthermore, since available information guiding kidney patients’ care is limited, new approaches are necessary.^5^ An important barrier to the development of new therapeutic approaches is represented by the limited understanding of molecular mechanisms underlying CKD, and the lack of therapeutic targets.

As highly metabolically active organ, kidney function is tightly dependent on mitochondria performance. Mitochondria are not only the cell powerhouse but also coordinate cellular adaptation to stressors, and regulate cell death, oxidative stress and cellular metabolism.^6^, ^7^ Therefore, mitochondrial damage and dysfunction have been associated to the pathophysiology of a broad spectrum of renal diseases, including kidney fibrosis development,^8^, ^9^, ^10^ CKD^11^, ^12^ and diabetic kidney disease.^13^

Axl, a member of the TAM family of receptor tyrosine kinases, is widely expressed in normal cells and tissues. Because of its function as a regulator of different physiological processes including cell survival, proliferation, migration and differentiation, Axl has been proposed as a promising treatment target for different malignancies.^14^, ^15^

A previous study from our group demonstrated the effectiveness of the Axl inhibitor bemcentinib in alleviating fibrosis development in a murine unilateral ureteral obstruction (UUO) model.^16^ This model is widely used to elucidate the pathogenesis of obstructive nephropathy and the mechanisms responsible for progressive renal fibrosis, and as a model to investigate fibrosis attenuating treatments.^17^ Furthermore, since it reflects the progression of acute kidney injury (AKI) to CDK, UUO provides an important model to study mitochondrial dysfunction in kidney diseases.^18^

Based on this background, in this study, we addressed the effects of‐Axl‐inhibitor bemcentinib on mitochondrial dysfunction induced by UUO, by investigating renal cell transcriptome and amino acid metabolism.

## 2 MATERIAL AND METHODS

### 2.1 Animals and sample collection

Animal handling and sample collection were previously described in full detail.^16^ Briefly, eight‐ to nine‐week‐old male C57Bl/6JOlaHSD mice were acquired from Envigo (Horst, the Netherlands) and kept and managed in the local animal facility at the Department of Biomedicine, University of Bergen, Norway. All surgeries were performed under general anaesthesia with isoflurane gas. Left ureter was identified through a subcostal incision and ligated with a silk ligature. Animals were divided into three groups: UUO model treated with bemcentinib diluted in vehicle (0.5% hydroxypropyl‐methylcellulose in 0.1% tween 80) (n = 6) or only with vehicle (n = 6) and SHAM‐operated (n = 4).

The drug was administered twice daily by oral gavage at a dose of 50 mg/kg (10 ml/kg), from one day before surgery to 14 days post‐surgery. Mice were sacrificed fifteen days post‐surgery and blood was collected by retro‐orbital method or cardiac puncture. Kidneys were also harvested, cut into transverse slices, fixed in formaldehyde and embedded in paraffin according to standard procedures. RNA was extracted from frozen murine kidney poles and sequenced on the Illumina HiSeq4000 platform. The mRNA sequencing data were processed in the RStudio environment, where reads were aligned and counted. Log 2 CPM data were obtained and fold changes were calculated.

### 2.2 Total gene expression analysis

An over‐representation analysis (ORA) was performed on the differential expression dataset using the ClusterProfiler package and the Reactome pathway repository,^19^ based on significantly expressed genes (FC ± 1.15, q < 0.05). A principal component analysis (PCA) was performed on the normalized log2 CPM expression data to evaluate the variability in the transcriptomics dataset. A loading plot from the first two (n = 2) principal components (PCs) was generated to elucidate which genes contributed most to the variance observed in PC 1 and PC2 and was visualized using the ggplot package.^20^ Differential expression for genes involved in selected pathways was visualized using Gene Ontology (GO)^21^ and the ggplot2 package. All analyses were performed using the R programming language. Sequencing data were published in a previous study from our group^16^ and are available in the repository Gene Expression Omnibus https://www.ncbi.nlm.nih.gov/geo/query/acc.cgi?acc=GSE123674.

### 2.3 Mitochondrial gene expression analysis

Normalized log2 CPM expression data were filtered based on the public gene database Mouse MitoCarta 2.0 (www.broadinstitute.org/pubs/MitoCarta), and PCA was performed on selected genes with statistically significant differential expression values (FC ± 1.5, q‐value < 0.05) to evaluate data variability. Loadings from the first n (n = 2) principal components (PCs) were extracted and variance was visualized using the ggplot package.^20^ Genes in each PC were ranked based on their loadings, and the 40 genes with the highest loading score in each PC were selected for hierarchical clustering using Euclidean distance and Ward2‐linkage, and visualized with ComplexHeatmap package.^22^

### 2.4 Mitochondrial DNA extraction and qPCR quantitation

Murine kidney tissue from FFPE blocks was cut into three 10‐μm sections and DNA was isolated using the QIAamp DNA FFPE Tissue Kit (Qiagen, catalogue number: 56 404). DNA quality and quantity were determined using the NanoDrop™ One/OneC Microvolume UV‐Vis Spectrophotometer (ThermoFischer Scientific, catalogue number: ND‐ONE‐W). All kits and equipment were used according to manufacturer's instructions. DNA was stored at 4℃. NADH dehydrogenase subunit 1 (MT‐ND1, Mm04225274_s1) and Ribosomal Protein Lateral Stalk Subunit P0 (Rplp0, Mm00725448_s1) TaqMan probes (ThermoFisher Scientific, catalogue number: 4 331 182) and LightCycler II 480 Master Mix (Roche Diagnostics GmbH) were used for quantitative PCR analysis. Cp values were acquired and analysed with a LightCycler II 480 thermocycler (Roche Diagnostics GmbH). Relative mitochondrial DNA abundance was calculated using the DeltaDeltaCt method, and Rplp0 as an endogenous reference for genomic DNA. Mean DeltaCt for all the SHAM samples was used as a calibrator.

### 2.5 Immunohistochemistry

Three‐micron‐thick formalin‐fixed paraffin‐embedded sections from ligated and non‐ligated murine kidneys were deparaffinated in xylene and rehydrated in descending concentrations of ethanol. Epitope retrieval was performed in target retrieval buffer (pH6, Dako) using a microwave oven, and endogenous peroxidase activity was quenched, by 10 minutes incubation with peroxidase‐blocking solution (Dako). Unspecific binding sites blocking was achieved by incubating sections with 10% normal goat serum in PBS (Dako) for 30 minutes. Sections were then incubated for 60 minutes with rabbit polyclonal anti‐TOMM20 (catalogue number ab186735, Abcam) or anti‐SDHB primary antibodies (catalogue number HPA002868, Sigma‐Aldrich) at a 1:500 dilution in antibody diluent with background reducing agent (Dako). Primary antibodies were labelled using polymer‐Horseradish peroxidase‐conjugated anti‐rabbit immunoglobulins (Envision+^®^ system, Dako). Signal was visualized using 3,3′‐diaminobenzidine (DAB, Dako) and sections were counterstained using haematoxylin (Dako), dehydrated and cover‐slipped using a non‐aqueous mounting medium. All reagents and kits were used according to manufacturer´s instructions. All immunohistochemical reactions were performed on the auto‐immunostainer intelpthFLX (BioCare) at room temperature. Digital 20X slides were created by scanning sections with ScanScope(TM) in the Department of Pathology at Haukeland University Hospital in Bergen, Norway. Digital slides were viewed in ImageScope (Aperio), and positive pixels were quantified using the colour deconvolution algorithm version 9.1 (Aperio, CA, USA) after adjusting the default parameters to each staining. Total percentage (%) of positive pixels was used as visualization parameter and statistics was performed by Graphpad Prism 8.

### 2.6 Western Blot

Protein extraction from mouse kidney tissues was achieved by using RIPA buffer (Sigma‐Aldrich, catalogue no. R0278) with the addition of complete protease inhibitor (Roche, catalogue no. 4693116001) and phosphatase inhibitor cocktail (Sigma‐Aldrich, catalogue no. P5726). Protein concentration was determined using Pierce BCA Protein Assay Kit (Thermo Scientific, catalogue no. 23225). Proteins were separated in Bolt 4‐12% Bis‐Tris Plus electrophoresis gels and transferred to nitrocellulose membranes using iBlot 2 System. Membranes were blocked with 5% BSA in PBS containing 0.1% Tween‐20 and then incubated overnight with rabbit polyclonal Anti‐TOMM20 antibody (Abcam, ab186735) 1:2000 dilution and mouse‐monoclonal Anti‐SDHB antibody (Abcam, ab14714) 4µg/mL. SeeBlue Plus2 Pre‐stained Protein Standard (Invitrogen, LC5925) was used to visualize protein molecular weight. The blots were washed three times with a wash buffer (PBS, 0.1% Tween‐20) and then incubated for 1 hour either with goat anti‐rabbit (Abcam, ab205718) or goat antimouse secondary (ab205719) HRP‐linked antibodies. The blots were washed again and developed using Pierce ECL Plus Western blotting substrate (Thermo Fisher). Chemo‐luminescence signals were assessed using ChemiDoc Imaging System (Bio‐rad). Densitometry analysis was performed using the ImageJ software.

### 2.7 Serum amino acids profile

Blood samples were collected in Microvette 500Z‐gel tubes (Sarsted, Germany, catalogue no. 201344) and centrifuged at 10000 *g* for 10 minutes to separate plasma, which was stored at −80℃. Briefly, plasma proteins were precipitated by adding 5‐Sulfosalicylic acid dihydrate (Sigma‐Aldrich) containing the internal standard norleucine (Sigma‐Aldrich) (1:4; V:V) to plasma (140‐160μL). Solutions were centrifuged for 10 minutes at 14000 *g* and supernatants were diluted 1:1 with lithium citrate buffer A‐1 (Sykam GmbH catalogue no. S000015). Qualitative and quantitative determinations of plasma free amino acids were performed by reverse‐phase high‐performance liquid chromatography with post‐column derivatization of amino acids with ninhydrin, by using a Sykam Automatic Amino Acid Analyzer S433 (Sykam GmbH, Germany, catalogue no. 1120001).^23^

Resulting data were exported as a table with molar concentration for each respective metabolite, and analysis was performed with the RStudio Environment [R Core Team (2019); RStudio Team (2015)]. Metabolites with more than 10% missing values were removed. Remaining metabolites were imputed using the minimum method. For multivariate analysis, data were autoscaled, and PCA and loadings were visualized using the ggplot package.^20^ Fold changes were calculated and a Welch test was applied to the log‐transformed dataset to calculate statistical differences between sample groups for each metabolite.

### 2.8 Statistics

Data are presented in dot plots (median/interquartile ranges) for the number of samples. Mann‐Whitney U test was used to assess statistical significance. Data were analysed and figures produced by Graphpad Prism 8. *P*‐values lower than .05 were considered significant.

## 3 RESULTS

### 3.1 Genome‐wide transcriptome analysis of UUO‐murine model treated with Bemcentinib

To investigate the effect of bemcentinib in the UUO model, we analysed the transcriptome of ligated and non‐ligated kidneys treated with or without bemcentinib. The expression of 11 239 genes was significantly different in ligated and non‐ligated kidneys, being 5809 up‐regulated and 5430 down‐regulated in ligated organs. Moreover, 5652 genes were differentially expressed in bemcentinib‐treated ligated (Bem‐L) compared to vehicle‐treated ligated (Veh‐L) kidneys, being 3103 up‐regulated and 2549 down‐regulated. Only 37 genes were differentially expressed in non‐ligated kidneys treated with or without bemcentinib (Table 1).

**TABLE 1:** Gene expression comparison. Significant up‐ and down‐regulated genes

GeNe expression	Bem‐L vs Bem‐UL	Bem‐L vs Veh‐L	Bem‐UL vs Veh‐UL	Veh‐L vs Veh‐UL
Up‐regulated	5462	3103	27	5809
No change	3894	8886	14 501	3299
Down‐regulated	5182	2549	10	5430

**FIGURE 1:**
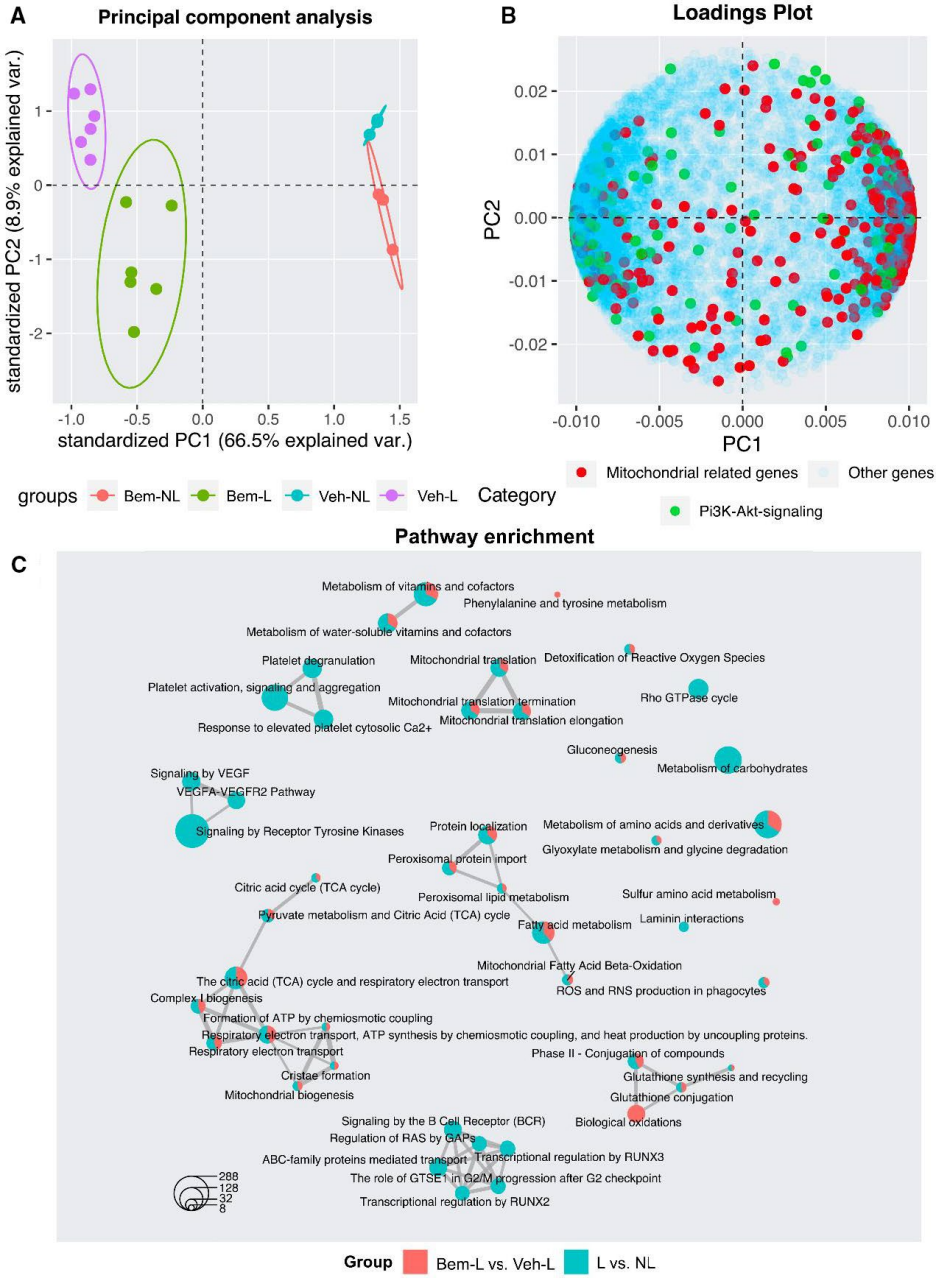
Multivariate analysis of transcriptomics data. A, Principal component analysis (PCA) performed on the transcriptomics dataset. Ligation is the major contributing factor (PC1: 66.5%) in separating samples, followed by treatment with bemcentinib (PC2: 8.9%). B, Loading plot with an overlay of mitochondrial‐related genes shows that changes in mitochondrial gene expression contribute to the variance observed in the PCA 3 Pathway enrichment shows the top 30 enriched terms from the Reactome Pathway Database. Larger nodes represent larger enrichment. Red refers to significant in Bem‐L compared to Veh‐L and blue between L vs NL

To obtain a general overview, we performed a PCA, which revealed that the majority of the variance (66.5%) in the dataset could be attributed to ligation (PC1), whereas bemcentinib treatment explained 8.9% of the variance (PC2), consistent with a clear difference between ligated and non‐ligated and treated or untreated kidneys (Figure 1A). The loadings from principal component 1 and 2 were visualized with an overlay of mitochondrial‐related genes from MitoCarta v2, to evaluate how changes in their expression affected the variance observed in the PCA. This analysis revealed that mitochondrial genes strongly contribute to the differences observed between ligated and non‐ligated kidneys on PC1, and that a majority of them had a positive weight (>0.075) towards non‐ligated kidneys. Interestingly, some loadings associated with mitochondrial‐related genes appeared to strongly contribute to the variance observed between bemcentinib‐treated and vehicle‐treated mice on PC2. Most of the weight (>0.1) from mitochondrial‐related loadings in PC2 pushed towards bemcentinib‐treated kidneys (Figure 1B).

For a more detailed interpretation of transcriptomic data, we performed a pathway over‐representation analysis (PORA) based on the Reactome Pathway Database. This analysis revealed that pathways related to tyrosine‐kinase signalling and transcription regulation (eg transcriptional regulation by RUNX2 and RUNX3 and regulation of RAS by GAPs) were significantly (q < 0.05, *P* < .05) enriched in ligated, compared to non‐ligated kidneys. Bemcentinib treatment mainly affected gene expression in pathways related to mitochondria, including oxidative stress (eg detoxification of reactive oxygen species, glutathione synthesis and recycling), oxidative phosphorylation (OXPHOS) (eg respiratory electron transport), amino acid metabolism (eg metabolism of amino acids and derivatives), citric acid cycle (TCA) and fatty acid metabolism (Figure 1C).

We then focused on genetic pathways affected by both ligation and bemcentinib treatment. Expression of genes related to pathways important for correct mitochondrial function, including citric acid cycle (TCA; GO:0006099), oxidative phosphorylation (OXPHOS; GO:0022900), response to ROS (GO:0034614), fatty acid oxidation (FAO; GO:0019395), urate acid metabolism (GO:0046415) and mitochondria‐related pathways such as glutathione metabolism (GO:006749), glycolysis (GO:0061621) and amino acid metabolism (GO:0006520), was, in general, reduced after ligation, but bemcentinib treatment increased it.

**FIGURE 2:**
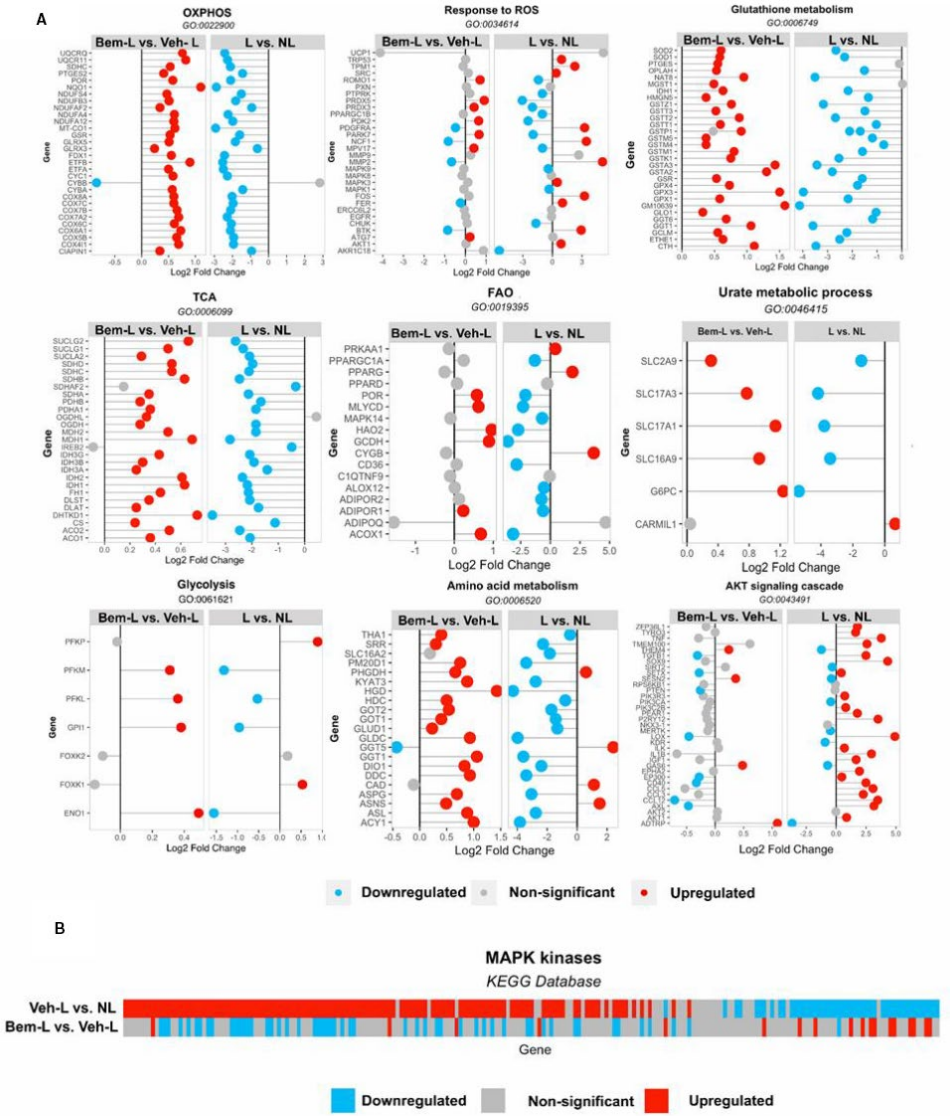
Univariate analysis of transcriptomics data. A, Differential expression of genes using GO‐terms. Data are consistent with a significant down‐regulation of the expression of genes involved in mitochondrial‐related processes after ligation and a significant reversal of these effects upon bemcentinib treatment, whereas PI3K/AKT signalling genes were up‐regulated after ligation but this effect was reversed by treatment with bemcentinib. B, Differential expression of genes related to MAPK signalling pathway using the KEGG database. Veh‐L vs NL genes are displayed at the top, whereas Bem‐L vs Veh‐L at the bottom. Up‐regulated genes (FC > 1.15, adj *P* value < 0.05) are shown in red, down‐regulated (FC < −1.15, adj *P* value < .05) in blue and non‐significantly affected in grey

In sharp contrast, AKT/PI3K signalling pathway genes were generally up‐regulated upon ligation, but bemcentinib largely reverted this transcription pattern (Figure 2A).

Since Axl is a tyrosine kinase signal transductor, we analysed differential expression of genes from MAPK‐related signalling cascades and pathways acquired from the KEGG database. This analysis revealed that a majority of genes in these pathways were also up‐regulated upon ligation, and bemcentinib treatment resulted in a considerable reversal of their transcription pattern (Figure 2B).

### 3.2 Effect of ligation and bemcentinib treatment in mitochondrial‐related gene expression

To further investigate how ligation and treatment with bemcentinib affected transcription of mitochondrial‐related genes, we performed several multivariate analyses on a data subset filtered through MitoCarta v2 public database.

**FIGURE 3:**
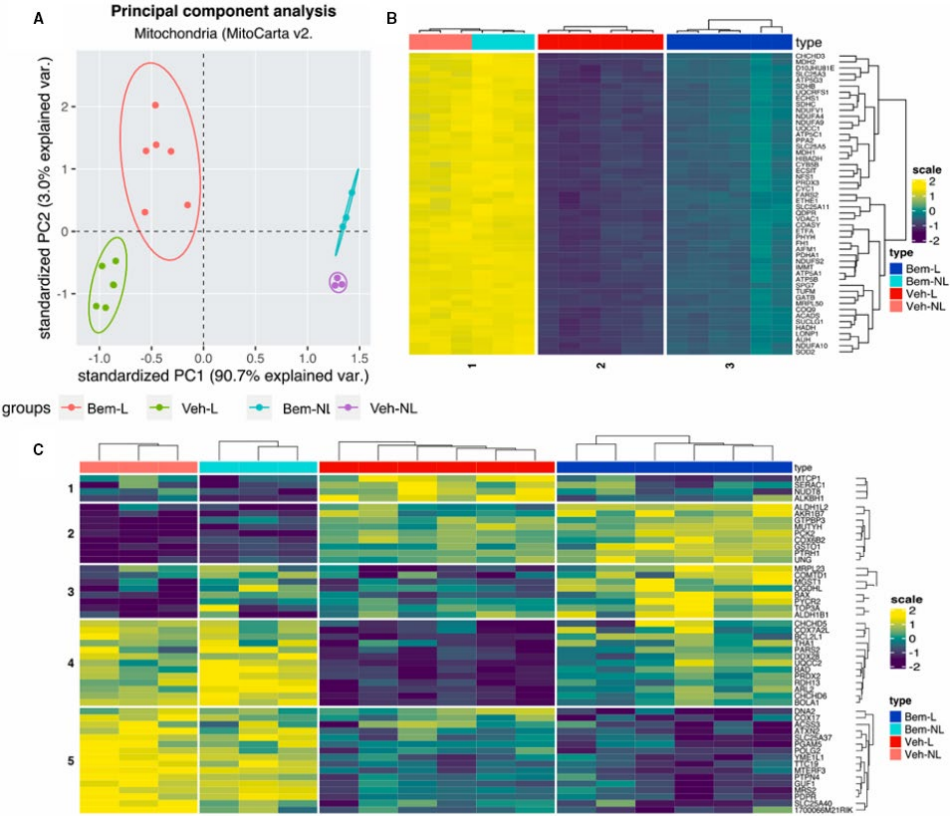
Multivariate analysis of mitochondria‐related genes. A, PCA based on the expression of significant genes (q < 0.05) filtered from MitoCarta v2. Database shows groups of samples clustering upon ligation and treatment. The variance seen in PC1 reflects ligation whereas PC2 represents the effect of bemcentinib treatment. B, Hierarchical clustering of top 50 loadings from PC1. C, Hierarchical clustering of top 50 loadings from PC2

A PCA on significant features revealed that the majority of variance (90.7%) in the dataset may be attributed to ligation, as shown in principal component 1 (PC1). The effect of bemcentinib, as explained by PC2, consisted in a 3.0% difference between the two treatment groups (Figure 3A).

We also performed hierarchical clustering on the top 50 genes with the highest loading in both PC1 and PC2 to investigate which genes most contributed to the variance seen in the PCA, and how ligation and treatment affected their expression. Top‐ranked genes in PC1 mostly showed a clear pattern of down‐regulation upon ligation, whereas bemcentinib treatment appeared to mildly reverse these effects (Figure 3B). However, in PC2, we observed a more diverse transcription pattern, identifying four distinct gene clusters where bemcentinib partially reversed the effects of ligation (Figure 3C).

### 3.3 Determination of mitochondrial biomass and dysfunction

Three approaches, two based on proteins and the other on DNA quantification, were used to determine if changes in mitochondrial gene expression could be associated with mitochondrial biomass alterations and dysfunction.

**FIGURE 4:**
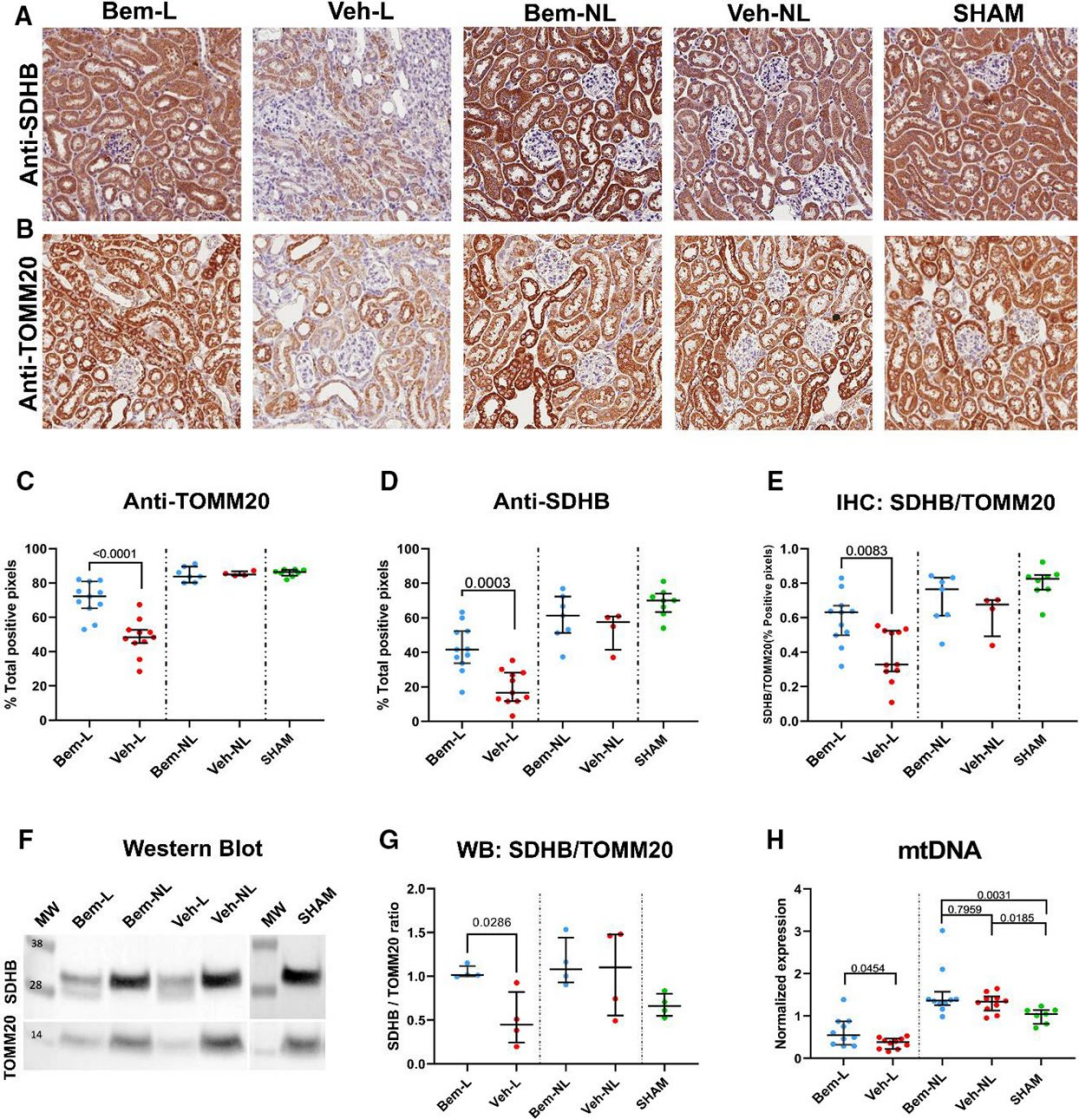
Visualization and quantification of TOMM20 and SDHB staining. Immunohistochemical analysis of sections from ligated and non‐ligated kidneys with or without bemcentinib treatment from male C57BI/6 mice after 14 d of ureteral obstruction for (A) SDHB and (B) TOMM20 protein expression. Quantitative analysis of positive pixels (%) with fibrotic tissue subtracted from quantification is provided for (C) TOMM20, (D) SDHB, (E) SDHB/TOMM20 ratio (paired samples). F, Western blot analysis of SDHB and TOMM20. G, Western blot protein quantification SDHB/TOMM20 ratio (paired samples). H, mtDNA quantification. All data were analysed by Mann‐Whitney U test

At the protein level, we determined two mitochondrial proteins: SDHB, located on the inner membrane of the mitochondria and participating in Citric Acid Cycle and electron respiratory chain, and TOMM20, a translocase located in the mitochondrial outer membrane by immunohistochemistry and western blot. Anti‐SDHB staining in ligated vehicle‐treated kidneys displayed a clearly weaker signal than in non‐ligated kidneys, and the same pattern was observed for anti‐TOMM20 (Figure 4A, B). Most importantly, bemcentinib treatment partially reverted these effects. Comparative positive pixels (%) quantification (Figure 4C, D) confirmed this data. However, the effect of Bemcentinib was not significant when measured by western blot (Figure 4F), probably because of the lower method sensitivity and because total protein was extracted including fibrotic tissue, that was removed from the immunohistochemistry quantification. Notably, with both techniques, SDHB/TOMM20 ratio was significantly lower in ligated kidneys and bemcentinib treatment also reverted this effect (Figure 4E and G).

At the genomic level, we quantified mitochondrial gene MT‐ND1, using nuclear gene Rplp0 as endogenous reference, to compare mitochondrial biomass within the samples.

This analysis revealed that ligated kidneys had significantly lower levels of mtDNA relative to nuclear DNA, as indicated by MT‐ND1 quantification, compared to non‐ligated kidneys, irrespective of treatment. However, bemcentinib‐treated ligated kidneys had significantly more mtDNA compared to vehicle‐treated ligated organs (relative levels to SHAM ± SD; Bem‐L: 0.639 ± 0.345; Veh‐L: 0.360 ± 0.126; *P* = .045). There was no significant difference between non‐ligated kidneys with or without bemcentinib treatment (Figure 4H).

### 3.4 Metabolomic profiles in bemcentinib and vehicle‐treated kidneys

**FIGURE 5:**
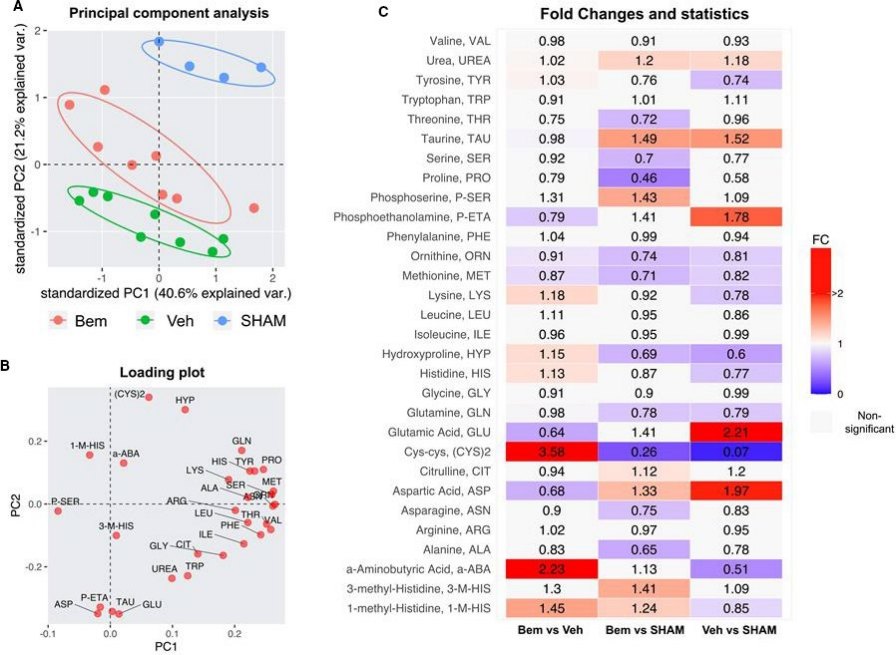
Multivariate analysis of serum amino acids and related metabolites. A, PCA shows groups of samples clustering according to ligation and treatment. The variance seen in PC2 reflects ligation and how treatment with bemcentinib appears to reverse the effects of ligation whereas PC1 represents differences between samples. B, Loading plot from PC2 shows how serum levels of modified amino acids separate different treatment groups whereas PC1 mainly consists of essential amino acids. C, Fold changes and statistics of serum metabolites

Considering the key role of mitochondria in amino acid metabolism, to complement our transcriptomics study, we performed a serum amino‐acid profile analysis in SHAM‐ and UUO‐operated animals with or without bemcentinib treatment. A PCA revealed that the majority of the variance could be attributed to within‐sample variation (PC1; 40.6% of explained variance), whereas the effect of ligation and bemcentinib treatment contributed to a clear separation between groups in PC2 (21.2%) (Figure 5A).

PC1 and PC2 loading visualization revealed that the majority of unmodified amino acids were driving the biological variation within samples seen in PC1, whereas the majority of the weight in PC2 was caused by modified amino acids (Figure 5B). Compared to SHAM‐operated animals, UUO‐operated animals were associated with an increase of TCA‐related amino acids glutamic acid and aspartic acid and a decrease in glycolysis and glutathione biosynthesis‐related amino acid cysteine. This effect was at least in part reversed by bemcentinib treatment. Fold change and significance of all the metabolites analysed in bemcentinib vs vehicle‐treated, bemcentinib vs SHAM and vehicle vs SHAM treatment are shown in Figure 5C.

## 4 DISCUSSION

In a previous study from our group, we found that tyrosine kinase receptor AXL is involved in the progression of renal fibrosis in a UUO murine model and that AXL inhibitor bemcentinib attenuates disease development.^16^ Mitochondrial dysfunction and oxidative stress have been demonstrated to play a role in the pathogenesis of renal fibrosis in both, humans and animal models.^24^, ^25^ In this study, we investigated the effects of Axl‐inhibitor bemcentinib on mitochondrial dysfunction associated with renal fibrosis.

The main function of mitochondria is to produce energy through respiration and molecular catalysis, thereby playing a central role in cell metabolism. Targeting defective mitochondria‐related pathways has also been proposed as a potential treatment of a variety of diseases, including different types of fibrosis,^26^, ^27^ diabetic kidney disease and CKD.^28^ Here, using RNA sequencing data from a murine UUO model,^29^, ^30^ we identified a pattern of dysregulated genes suggesting an impaired mitochondrial bioenergetics in ligated kidneys following 14 days post‐surgery, compared to non‐ligated kidneys. In general, mitochondria‐related genes were significantly down‐regulated in ligated compared to non‐ligated kidneys. However, this pattern was at least partially reverted in bemcentinib‐, compared to vehicle‐treated animals, thus indicating that bemcentinib has a potential beneficial effect on mitochondrial dysfunction occurring during renal fibrosis. Interestingly, unlike in ligated kidneys where bemcentinib had a clear effect, in non‐ligated kidneys, bemcentinib had little to no effect at the transcriptome level.

Mitochondrial homeostasis is tightly regulated and the disruption of the dynamic processes of mitochondrial biogenesis, fission/fusion and mitophagy impacts on renal injury and recovery.^31^ To compare mitochondrial biomass and dysfunction in our model, we used two methods. Firstly, we quantified mtDNA. Moreover, since mitochondria are dynamic organelles with a variable number of circular mtDNAs, we also analysed them at the protein level using two mitochondrial biomarkers, TOMM20 and SDHB. TOMM20 is a translocase located in the mitochondrial outer membrane that has an essential role in the specificity of mitochondrial protein import,^32^ whereas SDHB is part of the complex II of the respiratory chain located on the inner membrane of the mitochondrion linking citric acid cycle and oxidative phosphorylation, two critically important pathways in energy conversion. By using either approach, we found that mitochondria biomass was significantly decreased in ligated compared to non‐ligated kidneys, but bemcentinib significantly increased mitochondria biomass compared to vehicle‐treated animals.

A common by‐product of mitochondrial OXPHOS is represented by ROS, which are efficiently removed by different scavenging systems such as glutathione oxidation‐reduction cycle and superoxide dismutases (SODs). Increased ROS induces oxidative stress,^33^ associated with CKD and fibrosis progression.^11^, ^34^ We observed a down‐regulation of antioxidant genes upon ligation, but a positive effect of bemcentinib, that increased the expression of these genes.

Mitochondria play key roles in amino‐acid metabolism and are involved in both catabolic and anabolic processes.^7^ In particular, renal mitochondria critically contribute to nitrogen and amino‐acid homeostasis, and ammonia disposal, by renal deamidation of glutamine to glutamate (GLU).^35^ Furthermore, other studies in a UUO model in rats have revealed changes at the metabolomic profile levels in serum, tissue and urine.^36^, ^37^, ^38^, ^39^ To determine if bemcentinib had an effect in the metabolomic profile, we measured serum amino acids and other small metabolite levels comparing UUO with or without treatment and SHAM‐operated mice. Although some GLU derived from renal deamidation returns to the systemic circulation, previous studies suggest that the majority of GLU is converted to alanine (ALA) or further metabolized to alpha‐ketoglutarate (a‐KG), entering TCA cycle.^40^ In our study, bemcentinib treatment decreased GLU levels, whereas glutamine and ALA were not significantly affected. Furthermore, aspartate, which is synthesized through the transamination of TCA intermediate oxaloacetate, was also reduced in plasma upon treatment with bemcentinib. These results are in agreement with findings from similar metabolomics studies in UUO model and CKD patients, where increased GLU and ASP levels were observed in renal disease.^41^, ^42^ Also, serum levels of phosphoethanolamine (P‐ETA), involved in phospholipids metabolism, inhibiting mitochondrial respiration ^43^ and disrupting mitochondrial membrane potential,^44^ were decreased by bemcentinib treatment. In contrast, we did not observe any differences in levels of urea between the bemcentinib‐ and vehicle‐treated mice, suggesting an adaptation of hepatic production to compensate for the reduced renal capacity to dispose of urea through GLU‐GLN metabolism.

It should be noted that although it is widely used as a renal fibrosis model, UUO requires aggressive surgery that leads to a rapid interstitial inflammation at difference with the slow progression observed in humans.^17^, ^45^ Nevertheless, this model has proven useful both for the identification of biomarkers and for the development of new treatments.^46^, ^47^, ^48^ Furthermore, our study documents that bemcentinib was able to improve mitochondrial function at the molecular level despite the intrusiveness of the experimental method.

In conclusion, our data indicate that mitochondria and mitochondrial‐related pathways are dramatically affected by UUO surgery. Bemcentinib partially reverses the effects of UUO, without affecting non‐ligated kidneys or SHAM‐operated mice, and thereby qualifies as a promising treatment in kidney disease.

## CONFLICT OF INTEREST

This study was partly supported by BerGenBio ASA, which has also provided the bemcentinib. JBL declares ownership in BerGenBio ASA. GG is employed by BerGenBio ASA.

## AUTHOR CONTRIBUTIONS

**August Hoel:** Formal analysis (lead); Investigation (equal); Visualization (equal); Writing‐review & editing (equal). **Tarig Osman:** Conceptualization (supporting); Methodology (supporting); Project administration (equal); Writing‐review & editing (equal). **Fredrik Hoel:** Investigation (supporting); Methodology (supporting). **Hassan Elsaid:** Investigation (supporting); Writing‐review & editing (supporting). **Tony Chen:** Formal analysis (supporting); Writing‐review & editing (supporting). **Lea Landolt:** Investigation (supporting); Methodology (supporting); Writing‐review & editing (supporting). **Janka Babickova:** Investigation (supporting); Methodology (supporting). **Karl Johan Tronstad:** Methodology (supporting); Supervision (supporting); Validation (supporting); Writing‐review & editing (supporting). **James Lorens:** Funding acquisition (supporting); Supervision (supporting). **Gro Gausdal:** Conceptualization (supporting); Funding acquisition (supporting); Project administration (supporting). **Hans‐Peter Marti:** Conceptualization (equal); Funding acquisition (lead); Methodology (supporting); Project administration (lead); Supervision (equal); Writing‐review & editing (equal). **Jessica Furriol:** Conceptualization (lead); Formal analysis (supporting); Investigation (lead); Methodology (equal); Supervision (lead); Visualization (equal); Writing‐original draft (lead).

## Data Availability

Transcriptomics data analysed in this study were a re‐analysis of existing data, which are available at the repository Gene Expression Omnibus https://www.ncbi.nlm.nih.gov/geo/query/acc.cgi?acc=GSE123674. Any other data that support the findings of this study are available on request from the corresponding author.
